# Response to Ahmed G *et al.* concerning ‘Accuracy of estimates of serving size using digitally displayed food photographs among Japanese adults’

**DOI:** 10.1017/jns.2023.15

**Published:** 2023-02-17

**Authors:** Nana Shinozaki, Kentaro Murakami

**Affiliations:** Department of Social and Preventive Epidemiology, School of Public Health, The University of Tokyo, 7-3-1 Hongo, Bunkyo-ku, Tokyo 113-0033, Japan

Dear Editor:

We appreciate the comments by Ahmed G *et al.* on our recently published study entitled ‘Accuracy of estimates of serving size using digitally displayed food photographs among Japanese adults’^(1)^.

We agree with the comment on the importance of considering the amount of food consumed rather than served. As written in the Discussion section, we did not evaluate the validity of food photographs in estimating the amount of food consumed for hygienic reasons, which is a major limitation of this study. However, we are currently developing a web-based 24-h dietary recall system incorporating the food atlas. Using the 24-h meal recall system, we aim to further evaluate the validity of the food atlas in estimating actual food portions consumed.

We recognise that the accuracy of the estimates could be improved by providing several options for reporting portion sizes as employed in 24-h recall systems, such as Intake24^(2)^. In the Conclusion section, we wrote that combining food photographs and other methods, such as line diagrams and textual descriptions, would be useful for estimating portion sizes^(3–5)^. In the developing 24-h recall system, we would like to provide appropriate options for response methods.

Although not mentioned in the study, each participant was provided a unique identifier during their application for study participation, which was used in the serving and estimation sessions. Thus, there is no concern about the possibility of behavioural change because of the use of participants’ names in answering the web-based questionnaire.

Including eating disorder-related questions would be useful. Nevertheless, we asked the hunger level of each participant using a 5-point Likert scale in the serving session (see the Methods section). Consequently, there were no relative differences between estimated and true serving sizes for any food items among the hunger level categories (‘Association between participant characteristics and estimation error’ in the Results section).

Again, we thank Ahmed G *et al.* for the interest and comments on our study.
